# Portfolio analysis of NCI-funded implementation science grants studying sustainability of cancer control evidence-based practices

**DOI:** 10.1186/s43058-026-01039-1

**Published:** 2026-08-11

**Authors:** Margarita Correa-Mendez, David A. Chambers, Gila Neta

**Affiliations:** 1 Center for Global Health, National Cancer Institute, 9609 Medical Center Drive, Rockville, MD 20850 USA; 2 Division of Cancer Control and Population Sciences, National Cancer Institute, 9609 Medical Center Drive, Rockville, MD 20850 USA

**Keywords:** Implementation science, Sustainability, Cancer control

## Abstract

**Background:**

Sustainability is a key focus in implementation science given the global challenge of ensuring evidence-based practices (EBPs) deliver long-term benefits across community, public health, and clinical settings. We conducted a portfolio analysis of NCI-funded implementation science grants to describe the characteristics of funded applications that proposed to study the sustainability of cancer control EBPs and identify opportunities to advance the field.

**Methods:**

A systematic search using the National Institutes of Health (NIH) Query View Report tool identified NCI-funded implementation science grants with sustainability-focused aims awarded between fiscal years 2017 and 2023. Two coders independently reviewed full grant application materials to determine eligibility and extract information using a structured codebook developed, pilot-tested, and iteratively revised prior to full coding. Variables captured study objectives, proposed designs, sustainability measurement approaches, outcomes, implementation strategies, how sustainability was defined, and theories, models, or frameworks proposed.

**Results:**

Of 351 implementation science grants identified through the search strategy, 72 included at least one sustainability-focused aim and were included in the analysis. Most funded applications (*n* = 58, 81%) were awarded between 2020 and 2023. Only 4 proposed sustainability as the focus of all study aims. The majority examined sustainability determinants (*n* = 55, 76.4%), and only one proposed to evaluate the impact of an implementation strategy on sustainability. Funded applications focused primarily on cancer prevention (*n* = 54, 75%) and screening (*n* = 33, 45.8%). More than half (*n* = 39, 54.2%) did not provide an explicit definition for sustainability-related terms, and many used sustainability, maintenance, and sustainment interchangeably. Of the 72 funded applications, 64 (88.9%) used at least one theory, model, or framework, and 32 (44.4%) used two or more in combination.

**Conclusions:**

Our findings highlight opportunities to advance sustainability research across several areas, including the operationalization of sustainability concepts at the grant design stage, use of sustainability-specific frameworks and measurement tools, evaluation of implementation strategies designed to support sustainability of EBPs, and documentation of sustainability-relevant adaptations across diverse organizational, community, and health system contexts. Addressing these gaps can help to build a stronger evidence base for the sustainability of cancer control EBPs in the United States and globally.

**Supplementary Information:**

The online version contains supplementary material available at https://doi.org/10.1186/s43058-026-01039-1.

Contributions to the literature

- This study provides an overview of how sustainability is conceptualized, measured, and incorporated into National Cancer Institute (NCI)-funded implementation science grants focused on cancer control evidence-based practices.
- This analysis highlights opportunities to strengthen sustainability research, through clearer conceptualization of sustainability, broader use of sustainability-specific theories, models, frameworks, and measures, and greater emphasis on implementation strategies designed to support long-term sustainability.
- The findings identify opportunities for future sustainability research across the cancer control continuum to strengthen the long-term impact of evidence-based practices.

## Background

Enhancing understanding of how and under what conditions evidence-based programs, practices, policies and implementation strategies are sustained over time may increase their long-term impact across community, public health and clinical settings [1]. Sustainability remains an evolving and understudied area within the field of implementation science and has been described as “one of the most significant translational problems of our time” [1, 2]. Advancing the sustainability research agenda and highlighting the importance of considering sustainability throughout the planning, implementation and evaluation of research studies are priorities within the implementation science field [3]. Improving understanding of the multi level determinants that influence sustainability in complex and dynamic settings, developing and evaluating sustainability measures, and identifying implementation strategies designed to support sustainability are important areas of interest for researchers, donors, practitioners, and communities [4, 5].

There is a growing consensus in the field of implementation science around the definition of sustainability proposed by Moore et al., which describes sustainability as “after a defined period of time, the program, clinical intervention, and/or implementation strategies continue to be delivered and/or individual behavior change (i.e., clinician, patient) is maintained; the program and individual behavior change may evolve or adapt while continuing to produce benefits for individuals/systems” [6]. Other related terms include sustainment, defined as the continued use of an innovation [7], and maintenance, defined as the extent to which a program or policy becomes integrated into routine organizational practices [8]. As sustainment is the outcome of having maintained an EBP, in this paper we use the term sustainability to broadly encompass these terms.

Through the Dissemination and Implementation Research in Health program announcements (DIRH PAR), the National Institutes of Health (NIH) has supported a broad range of studies to generate evidence on implementation outcomes and implementation strategies relevant to diverse settings and stakeholder groups [9–11]. In the context of cancer control research, growing investment by the National Cancer Institute (NCI) in implementation science has been documented by prior portfolio analyses examining NCI-funded DIRH applications across the cancer care continuum [12, 13]. These analyses highlighted the need for more studies focusing on sustainability. Neta et al. reported that only 7 out of 71 NCI-funded DIRH grants awarded between 2006 and 2019 studied sustainability [12]. Similarly, an analysis examining how investigators conceptualized and planned for sustainability within NIH R01 grants (the primary investigator-initiated funding mechanism of the National Institutes of Health) found that out of 84 grants awarded between 2004 and 2016, 51 referenced sustainability-related concepts, but only 3 described the intent to study sustainability exclusively [14]. To build on these prior analyses, the current study examined NCI-funded implementation science grants awarded between 2017 and 2023 and expanded inclusion beyond R01 grants to include additional research funding mechanisms, including R37 awards and U-series cooperative agreements.

To examine the current landscape of sustainability research funded by the NCI, we conducted a portfolio analysis on NCI funded implementation science applications that include aims focused on sustainability of cancer control evidence-based practices (EBPs). The objectives of this portfolio analysis were to (1) Provide an overview of NCI-funded implementation science grants awarded by the NCI between the fiscal years (FY) 2017–2023 that include sustainability-focused aims; (2) Characterize the proposed approaches used to study, measure, and/or plan for sustainability of cancer control EBPs within funded applications and (3) Identify gaps and opportunities in the current NCI-funded portfolio to advance sustainability research.

## Methods

### Search strategy

We conducted a portfolio analysis of NCI-funded implementation science applications that included sustainability-focused aims. A systematic search was conducted using the NIH portfolio analysis platforms Query, View, Report (QVR) and iSearch to identify new and competing research grant awards funded by the National Cancer Institute (NCI) between fiscal years 2017 and 2023 that contained implementation science- and sustainability-related keywords in the title, abstract, specific aims, summary statements, and research strategy fields. Search terms were informed by prior NIH and NCI portfolio analyses [12–15] as well as the broader sustainability literature where relevant. Terms related to implementation science included “implementation science,” “implementation research,” “improvement science,” and “improvement research.” Sustainability-related terms included sustain*, continuation, maintenance, routinization, and institutionalization. Additional terms commonly used synonymously with sustainability were intentionally included to identify the broadest possible sample of funded applications that proposed sustainability-focused research.

The unit of analysis for this portfolio review was funded grant awards. The search included new and competing continuation research grants awarded or active between fiscal years 2017 and 2023 with NCI as the administrative center. The 2017–2023 time period was selected to extend prior analyses examining sustainability-focused NIH-funded research [14]. Resubmissions and competing renewals were treated as separate awards if they received independent funding decisions during fiscal years 2017–2023. No duplicate records were identified through the search process. Linked phases or mechanisms associated with the same overall project (e.g., UG3/UH3 phased awards) were reviewed independently and included only if the funded phase met eligibility criteria for implementation science and sustainability-focused aims.

**Fig. 1:**
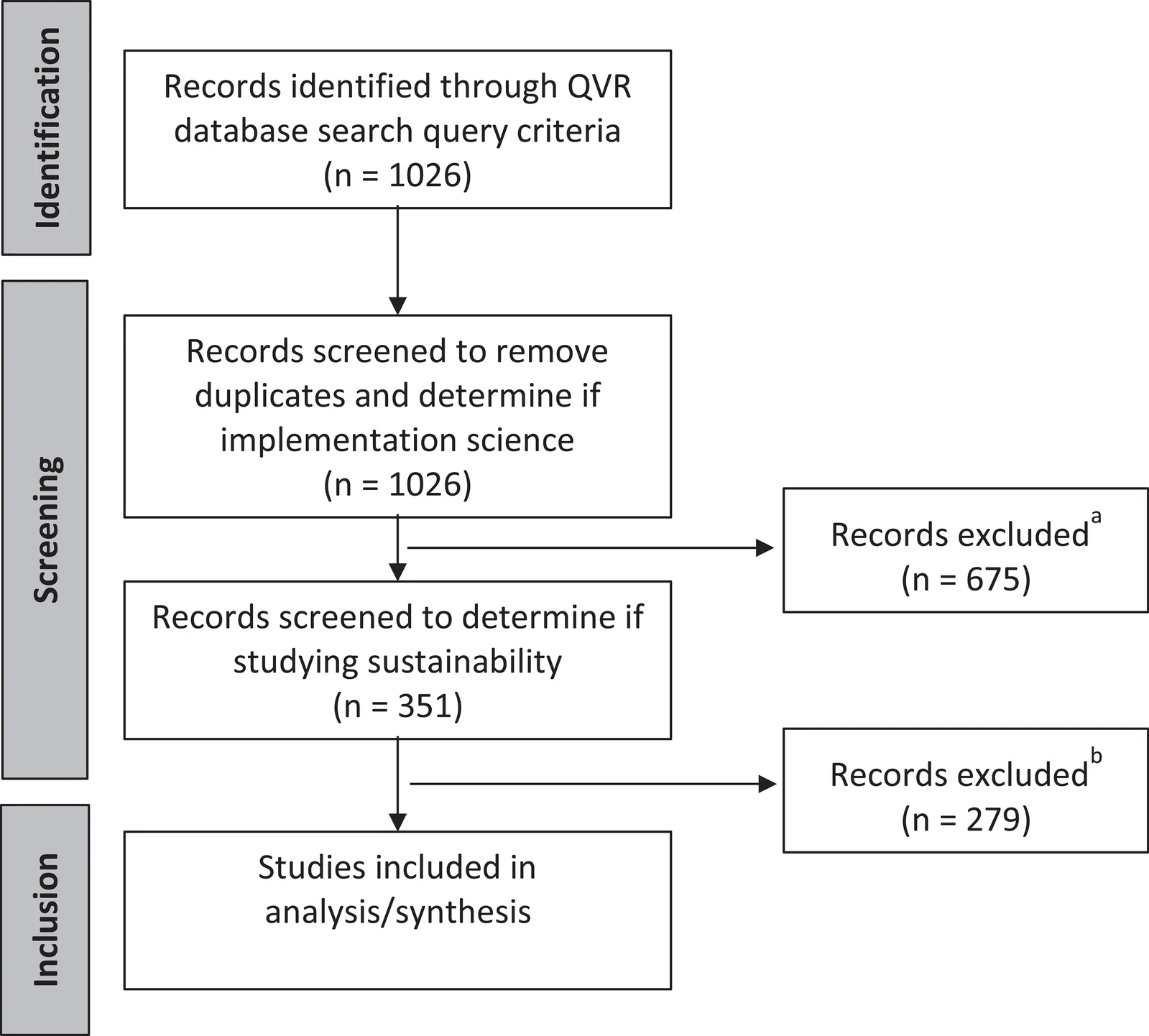
Flow diagram of the multi-step selection process used to identify the final sample of grants included in the portfolio analysis. ^a^ Exclusion reasons: not implementation science studies, used D-, F-, K-, SC or T-series funding mechanisms; ^b^ Exclusion reason: Did not have at least one aim focused on sustainability

The titles and abstracts of applications identified through the search strategy were independently reviewed by two coders (MCM and GN) to determine if they were implementation science studies eligible for coding and analysis. Applications were eligible if they were funded through R-series (research project grants) or U-series (cooperative research agreement) mechanisms and proposed at least one aim focused on sustainability of a cancer control EBP. To capture the range of concepts used in sustainability research, applications were included if they referenced sustainability, sustainment, maintenance, continuation, routinization, or institutionalization of a cancer control EBP. Applications were excluded if they: (1) did not propose implementation research, (2) did not include at least one sustainability-focused aim related to a cancer control EBP, (3) were duplicate records identified during screening, or (4) used D-series (research resources), F-series (fellowship), K-series (career development), SC-series (research-related infrastructure), or T-series (institutional training) funding mechanisms. Compared with the prior NIH sustainability analysis [14], the current review expanded the fiscal year range, extended eligibility to additional funding mechanisms (e.g., U-series cooperative agreements and R37 MERIT research awards), and incorporated review of full grant application materials, including research strategies. Figure 1 shows the Preferred Reporting Items for Systematic Reviews and Meta-Analyses (PRISMA) 2020 Statement [16] flow diagram as adapted to this portfolio analysis, documenting the multi-step screening process followed to identify the final sample of grants included in the analysis.

### Codebook and coding process

A codebook was developed by the authors to abstract relevant information from the final sample of funded applications included in the analysis. Development of the codebook was informed by the sustainability literature, prior NIH and NCI portfolio analyses, and feedback from sustainability researchers (Additional File 1). The codebook was piloted on a subset of 20 funded applications independently reviewed by two coders (MCM and GN) to refine coding definitions and promote consistency in coding. During the pilot phase, discrepancies were discussed and the codebook was iteratively revised to clarify variable definitions and coding procedures prior to full coding. All funded applications were independently reviewed by two coders, with discrepancies resolved through discussion and consensus reconciliation. Because coding decisions were finalized through consensus as part of the iterative coding process, formal kappa statistics were not calculated. The final version of the codebook was then applied to the remaining funded applications using the NIH internal platform iSearch. Variables included in the codebook captured key characteristics of the proposed studies, including grant descriptives (e.g., cancer type, area of focus along the cancer care continuum, intervention content area, study location, setting characteristics), study objectives, proposed study designs and analytic approaches related to sustainability, proposed approaches for sustainability measurement, sustainability outcomes, strategies proposed to support sustainability, contextual levels assessed in relation to the sustained use of the EBP, definitions of sustainability, and theories, models, or frameworks referenced within the application. Two authors (MCM and GN) independently coded the specific aims and research strategies of each grant using the iSearch platform. A third co-author (DC) reviewed the codebook, provided feedback throughout the coding process and addressed coding discrepancies as needed.

## Results

### Funding characteristics of included grants

Of the 1,026 grants retrieved through the search strategy, 351 were identified as implementation science grants, of which 72 proposed at least one sustainability-focused aim related to a cancer control EBP and were included in the final analysis. Table 1 shows the funding characteristics of these 72 implementation science grants on sustainability funded between fiscal years 2017 and 2023. Most funded applications (*n* = 58, 81%) were awarded between 2020 and 2023. These counts reflect absolute numbers of funded grants. Fifty-one applications (71%) were research project grants (R01 and R37 MERIT research awards), sixteen (22%) were cooperative agreements (U01 and UM1 mechanisms involving substantial programmatic involvement from NIH) and five (7%) were UG3 exploratory and developmental phased research awards. Out of the 72 funded applications included in the analysis, 20 (28%) involved research conducted in low- or middle-income countries (LMICs), based on the World Bank country income classification system current at the time of analysis [17]. Because country income classifications may change over time, these classifications may not reflect countries income status at the time individual grants were awarded.

**Table 1:** Funding characteristics of NCI-funded implementation science grants with sustainability-related aims (*N* = 72)

	Frequency	Percent
**Year Awarded**		
2023	9	12.5
2022	19	26.4
2021	17	23.6
2020	13	18.1
2019	3	4.2
2018	8	11.1
2017	3	4.2
**Funding mechanism**		
R01	47	65.3
U01	15	20.8
UG3	5	6.9
R37	4	5.6
UM1	1	1.4
Research in LMIC*		
Yes	20	28.0
No	52	72.0

*Low- or middle-income Country

### Cancer research characteristics

We characterized the study topic areas described in the funded applications, including the area of focus along the cancer control continuum, cancer type, and content area of the EBP (Table 2). Most funded applications proposed aims focused on cancer prevention (*n* = 54, 75%) while thirty-three (45.8%) focused on screening and twenty-three (32%) included aims related to survivorship-focused EBPs. Fewer funded applications proposed to study the sustainability of diagnosis-related (*n* = 3, 4.2%) and treatment-related EBPs (*n* = 12, 16.7%). The majority of funded applications (*n* = 42, 58.3%) did not focus on a specific cancer type as the EBPs described had broader applications across cancer prevention and control. Among funded applications focused on a specific cancer type, cervical cancer (*n* = 14, 19.2%) and colorectal cancer (*n* = 10, 13.9%) were the most commonly represented. Funded applications addressed several cancer content areas, with screening (*n* = 24, 33.3%), tobacco control (*n* = 16, 22.2%), and HPV vaccination (*n* = 10, 13.9%) representing the most common areas of focus. Among funded applications conducted in LMIC settings, cervical cancer screening, HPV vaccination and tobacco control were the most commonly proposed cancer content areas. The full list of content areas is described in Table 2.

**Table 2:** Cancer characteristics of the evidence-based practices described in NCI-funded implementation science grants (*N* = 72)

	Frequency	Percent
Cancer Continuum*		
Prevention	54	75.0
Screening	33	45.8
Diagnosis	3	4.2
Treatment	12	16.7
Survivorship	23	31.9
Cancer Type*		
Not specified	42	58.3
Cervical cancer	14	19.4
Colorectal Cancer	10	13.9
Breast Cancer	6	8.3
Lung Cancer	3	4.2
Skin Cancer	2	2.8
Ovarian Cancer	2	2.8
Bladder Cancer	1	1.4
Lymphoma	1	1.4
Cancer content area*		
Screening	24	33.3
Tobacco control	16	22.2
HPV vaccination	10	13.9
Survivorship care	8	11.1
Genetic services	5	6.9
Physical activity	3	4.2
Nutrition	2	2.8
Sun safety	1	1.4

*Responses not mutually exclusive

### Characteristics of implementation science grants with sustainability-focused aims

The analysis of NCI-funded implementation science grants with sustainability-focused aims provided insights into the objectives, conceptualizations, and proposed study designs for investigating the sustainability of cancer control EBPs (Table 3). Fifty-six funded applications (77.8%) proposed aims focused on sustainability, thirty-five (48.6%) referenced maintenance and ten (13.9%) referenced sustainment. These terms were not mutually exclusive and were used interchangeably in some applications. Definitions of sustainability-related terms varied across funded applications (Supplemental File 2) and more than half of funded applications (*n* = 39, 54.2%) did not provide an explicit definition for sustainability, maintenance, or sustainment. Among funded applications that stated a definition, the most common conceptualization focused on continued delivery or implementation of the EBP over time (*n* = 10, 13.9%), followed by institutionalization or integration within routine organizational practice (*n* = 8, 11.1%). The most common study objectives were to examine determinants of sustainability (e.g., barriers and facilitators) (*n* = 55, 76.4%) and measure the sustainability (*n* = 40, 55.6%) of EBPs. Among the applications that proposed to measure sustainability, four planned to use the Program Sustainability Assessment Tool (PSAT) [18], three the Clinical Sustainability Assessment Tool (CSAT) [4], four the Sustainment Measurement System Scale [19] and one the Provider REport of Sustainment Scale (PRESS) [20]. The remaining 28 funded applications proposed study-specific measures related to the continued delivery of the EBPs. Among funded applications that proposed to examine contextual factors related to sustainability, the organizational (*n* = 57, 79.2%) and provider-level (*n* = 40, 55.6%) context were most commonly assessed. Fewer funded applications included aims focused on examining the policy context as relevant to the sustainability of the EBP (*n* = 7, 9.7%). The most commonly proposed study designs were mixed methods (*n* = 26, 36.1%) and qualitative approaches (*n* = 25, 34.7%).

The most commonly proposed sustainability outcomes were continued program activities or core elements of the intervention (e.g. continued program implementation) (*n* = 31, 43.1%) and maintaining organizational practices, procedures, and policies initiated during implementation (institutionalization) (*n* = 17, 23.6%). Less commonly represented sustainability outcomes included documenting adaptations to intervention components (*n* = 5, 6.9%) and maintaining community-level partnerships or community capacity for collaboration (*n* = 1, 1.4%). Among funded applications that specified follow-up timepoints for assessing sustainability-related outcomes, the most common proposed timeframes were 12 to 18 months (*n* = 28, 39%) and 24 months after initial implementation. Twenty-two grants did not specify sustainability follow-up timepoints.

Various theories, models and frameworks were proposed across the funded applications. The most commonly referenced framework was Reach, Effectiveness, Adoption, Implementation, Maintenance (RE-AIM) [8] (*n* = 37, 51.4%). Frameworks with explicit sustainment or sustainability domains were less commonly proposed and included Exploration, Preparation, Implementation, Sustainment (EPIS) [7] (*n* = 7, 9.7%), the Practical Robust Implementation and Sustainability Model (PRISM) [21] (*n* = 6, 8.3%), and the Dynamic Sustainability Framework (DSF) [22] (*n* = 5, 6.9%). Of the 72 funded applications, 64 (88.9%) proposed use of at least one theory, model, or framework, and 32 (44.4%) proposed use of two or more frameworks in combination.

**Table 3:** Characteristics of sustainability research proposed in NCI-funded implementation science grants (*N* = 72)

	Frequency	Percent
Terms used*		
Sustainability	56	77.8
Maintenance	35	48.6
Sustainment	10	13.9
Continuation	1	1.4
Study Objectives*		
Examine determinants (barriers/facilitators)	55	76.4
Measure sustainability	40	55.6
Assess costs and benefits	8	11.1
Evaluate the impact of an implementation strategy on sustainability	1	1.4
Methodological (develop tools for sustainability research)	1	1.4
Study design*		
Mixed methods	26	36.1
Qualitative	25	34.7
Experimental	19	26.4
Observational	17	23.6
Quasi-experimental	2	2.8
Systems science	1	1.4
Level of context examined*		
Organization	57	79.2
Provider	40	55.6
Patient	20	27.8
Community	10	13.9
Policy	7	9.7
Sustainability outcomes*		
Continuing program activities or core elements of the intervention	31	43.1
Maintaining organizational practices, procedures, and policies started during implementation	17	23.6
Adaptation of intervention components	5	6.9
Maintaining community-level partnerships or coalitions and community capacity for collaboration	1	1.4
Not specified	21	29.2
Follow-up*		
6 months or less	3	4.2
6–12 months	10	13.9
12–18 months	28	38.9
18–24 months	5	6.9
24 + months	25	34.7
Not specified	22	30.6
Theory/Model/Framework*		
Reach Effectiveness Adoption Implementation Maintenance (RE-AIM)	37	51.4
Consolidated Framework for Implementation Research (CFIR)	15	20.8
Proctor’s Implementation Outcomes Framework	11	15.3
Exploration, Preparation, Implementation, Sustainment (EPIS)	7	9.7
Practical Robust Implementation and Sustainability Model (PRISM)	6	8.3
Dynamic Sustainability Framework (DSF)	5	6.9
PARIHS	3	4.2
Interactive Systems Framework	1	1.4

*Responses not mutually exclusive

### Characteristics of sustainability research in global cancer control settings

Of the 72 funded applications included in the analysis, 20 (28%) proposed research in low- and middle-income countries (LMICs), including Senegal, Kenya, Vietnam, Peru, India, Lebanon, Nigeria, Zambia, Botswana, Mali, and Uganda. Other study settings included Puerto Rico as well as one multi-country study spanning up to 18 countries. Among these funded applications, cervical cancer screening, HPV vaccination, tobacco control and survivorship care represented the most commonly proposed cancer content areas. Most funded applications proposed to examine factors related to sustainability, the majority at the provider and organizational level. Twelve of the 20 funded applications proposed a specific aim focused on measuring maintenance, sustainment, or sustainability. Proposed approaches included the use of PSAT [18], CSAT [4], subscales of the Sustainment Measurement System Scale (SMSS) [19] and the Provider Report of Sustainment Scale (PRESS) [20]. Notably, all funded applications in LMIC settings proposed use of at least one TMF including: Sustainment Measurement System Scale [19], Public Health Capacity for Sustainability Framework [21], Dynamic Sustainability Framework [22], PSAT [18], CSAT [4], COM-B [23], INSPIRE [24], ot the Dynamic Adaptation Process (DAP) [25] integrated into the EPIS framework [7]. In some cases, these FWs were often proposed to be used in combination with RE-AIM [8], EPIS [7] or Proctor’s Implementation Outcomes Framework [26].

## Discussion

The purpose of this portfolio analysis was to describe NCI-funded implementation science grants with sustainability-focused aims and identify opportunities to advance sustainability research. The analysis identified a larger number of funded implementation science applications awarded between 2020 and 2023 that included at least one aim focused on sustainability of EBPs across the cancer continuum. The greater number of funded applications with sustainability-focused aims may partially reflect changes in NIH and NCI Dissemination and Implementation Research in Health (DIRH) program announcements over time, as sustainability became an explicit area of emphasis in DIRH funding opportunity reissuances [12]. Additionally, the broader increase in NCI-funded implementation science applications highlighted in prior portfolio analyses [12, 13] may have also contributed to the larger number of sustainability focused-applications identified in the current review. Because this portfolio analysis examined absolute counts rather than proportional trends relative to all eligible implementation science applications, these findings should be interpreted cautiously and not as evidence of a normalized increase in NCI support over time.

The number of funded applications with sustainability-focused aims may also reflect the growing recognition within the field of the importance of understanding how EBPs can be sustained, adapted and integrated within real-world settings over time to support long-term patient, organizational and population health benefits. However, only 4 of the 72 funded applications included in this portfolio analysis proposed research aims focused exclusively on sustainability of cancer control EBPs across all study aims. This observation is consistent with the Mascarenhas Johnson et al. analysis of NIH R01 grants, in which only 3 out of 51 NIH RePORTER profiles proposed to study sustainability exclusively [14]. Similar patterns have been described in systematic reviews of sustainability research, where sustainability is often addressed after an EBP has been designed, funded, and implemented rather than explicitly planned for at the outset of implementation [27]. Funded applications included in this portfolio were concentrated in cancer prevention and screening EBPs. This pattern may reflect the longer implementation history and more established evidence base of many prevention and screening interventions, which may make them particularly well suited for examining questions related to sustainability. In contrast, fewer funded applications proposed sustainability-focused aims related to diagnosis and treatment EBPs, highlighting potential opportunities to expand sustainability research across other areas of the cancer control continuum. Collectively, these findings suggest opportunities for future implementation research to incorporate sustainability planning throughout the design and conduct of studies.

Sustainability-focused aims within funded applications primarily emphasized examining determinants of sustainability at the organizational and provider levels, reflecting growing recognition that sustainability is shaped not only by EBP effectiveness, but also by health system, leadership, financing, workforce, and community dynamics that evolve over time [22, 27]. Although fewer funded applications explicitly proposed examining community-level determinants of sustainability, this may partially reflect the level of detail provided within grant application materials, as community engagement may have been embedded within broader implementation activities that were not fully specified. The literature in the field emphasizes that meaningful community engagement and long-term partnerships are foundational for sustainability and that greater attention to identifying, strengthening, and leveraging existing assets within individuals and communities is important to increase the likelihood EBPs are sustained [28]. These findings highlight continued opportunities for sustainability research to more explicitly examine how community partnerships, local norms and values, engagement of key partners, and broader socio-cultural contexts influence sustainability of EBPs across settings.

The limited number of funded applications proposing to evaluate the impact of implementation strategies explicitly designed to support sustainability of EBPs represents an important gap in the current portfolio, consistent with prior literature suggesting that studies evaluating sustainment or sustainability-focused strategies remain limited [29]. It is important to note that some sustainability-focused strategies may not have been fully specified within the grant application materials reviewed. These findings reinforce opportunities identified in the sustainability literature for studies that more explicitly identify, operationalize, and evaluate implementation strategies that align with local priorities and resources, while building evidence on the feasibility, acceptability, and impact of these strategies across diverse settings and contexts and identifying those most effective in supporting long-term sustainability [2, 28]. Existing resources, including sustainment-focused adaptations of the ERIC strategy taxonomy, may help guide the development, testing, and application of sustainability-focused implementation strategies; however, their impact on sustainment outcomes requires further empirical evaluation [30].

The variability and interchangeable use of sustainability-related terminology across funded applications reflects broader conceptual challenges within implementation science sustainability research, consistent with observations in the broader implementation science literature [29, 31]. A subset of funded applications drew on established definitions or sustainability-focused frameworks to anchor their conceptual approach, suggesting growing recognition of the importance of conceptual clarity in sustainability research. Clearer operationalization of sustainability concepts at the grant design stage, including explicit definitions and consistent application of terminology throughout the research strategy, may improve comparability of sustainability research across studies and settings. Because this analysis was based on funded applications and proposed research strategies, the extent to which definitions were explicitly operationalized or consistently applied in the research ultimately conducted may differ from what was described in the application materials reviewed.

Study-specific sustainability measures proposed within funded applications primarily focused on delivery-related outcomes rather than intervention outcomes. Although relatively few funded applications proposed to use published sustainment or sustainability measures such as PSAT, CSAT, Sustainment Measurement System Scale or PRESS, sustainability researchers have emphasized the importance of robust and relevant measures to advance understanding of sustainability and sustainability determinants across diverse settings [5]. Opportunities remain to further evaluate the applicability and validity of sustainability measures across diverse settings. Reviews of sustainability measurement tools have noted that many available measures were developed and psychometrically evaluated primarily in U.S.-based settings, potentially limiting cross-cultural validity and assessment of broader contextual determinants of sustainability [5]. The funded applications in this portfolio that proposed use of sustainability measures across diverse implementation settings, including LMIC contexts, present important opportunities to further examine sustainability measurement across health systems and implementation contexts. Future analysis of resulting publications and outcomes from funded applications may contribute important evidence regarding the feasibility, applicability, validity and needed adaptations of sustainability measures across diverse implementation, organizational, and health system contexts.

Relatively few funded applications proposed efforts to document adaptations to intervention components or strategies intended to maintain community-level partnerships and collaborative capacity, although these activities may not have been fully specified within grant application materials. As described by Shelton et al. (2018), sustainability outcomes extend beyond continued implementation to include adaptation, maintenance of organizational practices, sustained partnerships, and diffusion across settings [2, 31–33]. With increasing recognition that sustainability is a dynamic process, documenting adaptations across organizational, socio-cultural, community, and health system contexts may help advance sustainability research and improve applicability of findings across diverse settings [22].

Consistent with prior analyses of NIH implementation science grant applications [14], frameworks with explicit sustainment or sustainability constructs, including EPIS, PRISM, and the Dynamic Sustainability Framework, were less frequently represented. Other sustainability-focused frameworks, including the Framework by Shediac-Rizkallah and Bone [34], the Integrated Sustainability Framework [2], and regionally developed frameworks such as the Conceptual Framework of Sustainability of Interventions implemented in Sub-Saharan Africa [27], were not identified within the funded applications included in this analysis. Notably, funded applications conducted in LMIC settings proposed use of sustainability-focused frameworks or measures in combination with broader implementation science frameworks such as RE-AIM [8], EPIS [7], or Proctor’s Implementation Outcomes Framework [26]. Sustainability-focused frameworks may help operationalize sustainability concepts, support identification of relevant sustainability determinants, outcomes, and measures and improve understanding of how sustainability evolves across dynamic organizational, community, and health system contexts over time. Additional frameworks in the field, including FRAME [35] and FRAME-IS [36], may also support the systematic documentation of adaptations needed to sustain EBPs over time across organizational and health system contexts. Together with prior literature, these findings suggest continued opportunities to empirically evaluate sustainability-focused frameworks, measures, and adaptation approaches across diverse cancer control EBPs and implementation settings [2].

### Strengths and limitations

This portfolio analysis had several strengths, including the expanded fiscal year range (2017–2023), the inclusion of additional funding mechanisms beyond R01 grants, the use of dual independent coders, and a structured codebook informed by the sustainability literature, prior NIH and NCI portfolio analyses including prior sustainability-focused work [14], and feedback from sustainability researchers. As an internal NIH project, coders had access to full grant application materials, including research strategies and specific aims, enabling more detailed characterization of proposed sustainability research than prior analyses that relied on abstracts or NIH RePORTER profiles alone. The codebook captured a broader range of sustainability-related variables than previously used in portfolio analyses, including sustainability outcomes, implementation strategies designed to support sustainability, approaches to sustainability measurement, how sustainability was conceptualized and defined, and theories, models, and frameworks proposed. The inclusion of funded applications involving LMIC settings further adds global relevance to the findings and contributes to understanding of how sustainability research is being conducted across diverse health systems.

This analysis has several limitations. Although a broad range of search terms was used, some relevant grants may not have been captured. In addition, this portfolio analysis was restricted to NCI-funded grants and therefore does not reflect sustainability research funded by other NIH institutes, other U.S. federal agencies, or non-U.S. funding organizations. This analysis also examined absolute counts of funded grants over time and did not assess the proportion of sustainability-focused grants relative to all eligible NCI-funded implementation science grants in each fiscal year. Findings should not be interpreted as evidence of a normalized increase in NCI support for sustainability research over time. Another limitation is that the analysis was based on funded grant applications and proposed research strategies rather than the research ultimately conducted or published. As such, findings reflect investigators planned study aims, proposed measures, and intended research approaches, which may differ from study implementation or findings reported in subsequent publications. Certain sustainability-related variables may not have been fully detailed in application materials, which could contribute to undercounting these activities. Finally, despite piloting the codebook on a subset of funded applications and developing thorough coding definitions, coding remains subjective, though this was mitigated by dual independent coding and consensus reconciliation throughout.

## Conclusions

This portfolio analysis advances understanding of how NCI-funded implementation science grants have proposed to study the sustainability of cancer control EBPs. Opportunities remain to advance sustainability research across several areas, including clearer operationalization and consistent definition of sustainability concepts at the grant design stage, broader use of sustainability-specific frameworks and measurement tools, more explicit evaluation of implementation strategies designed to support sustainability of EBPs, and greater attention to documenting adaptations relevant to sustainability across diverse organizational, community, and health system contexts. Funded applications involving LMIC settings demonstrated greater use of sustainability-focused frameworks and measurement tools in combination with broader implementation science frameworks, offering important lessons for how sustainability can be operationalized across diverse health systems. Continued investment in sustainability research and collaboration with multi-level health system, organizational, and community partners can help maximize the long-term impact of NIH investments and address the cancer burden in the United States and globally.

## Supplementary Information

Below is the link to the electronic supplementary material.

Supplementary Material 1

Supplementary Material 2

## Data Availability

Full copies of the grants from which these data were generated are considered confidential per federal regulations and NIH grant policies and are not available upon request or available in the public domain.

## Abbreviations

IS: Implementation science
CFIR: Consolidated Framework for Implementation Research
CSAT: Clinical Sustainability Assessment Tool
DSF: Dynamic Sustainability Framework
EBP: Evidence-based practice
EPIS: Exploration preparation implementation sustainment
HPV: Human papillomavirus
LMIC: Low- and middle-income country
NCI: National Cancer Institute
NIH: National Institutes of Health
PRISMA: Preferred Reporting Items for Systemic Reviews and Meta-Analyses
QVR: Query, View, and Report
PSAT: Program Sustainability Assessment Tool
RE-AIM: Reach Effectiveness Adoption Implementation Maintenance

## References

[1] Proctor E, Luke D, Calhoun A, McMillen C, Brownson R, McCrary S, Padek M. Sustainability of evidence-based healthcare: research agenda, methodological advances, and infrastructure support. Implement science: IS. 2015;10:88. 10.1186/s13012-015-0274-5 PMC4494699 26062907

[2] Shelton RC, Cooper BR, Stirman SW. The Sustainability of Evidence-Based Interventions and Practices in Public Health and Health Care. Annu Rev Public Health. 2018;39:55–76. 10.1146/annurev-publhealth-040617-014731 29328872

[3] Shelton RC, Chambers DA, Glasgow RE. An Extension of RE-AIM to Enhance Sustainability: Addressing Dynamic Context and Promoting Health Equity Over Time. Front Public Health. 2020;8:134. 10.3389/fpubh.2020.00134 PMC7235159 32478025

[4] Malone S, Prewitt K, Hackett R, Lin JC, McKay V, Walsh-Bailey C, Luke DA. The Clinical Sustainability Assessment Tool: measuring organizational capacity to promote sustainability in healthcare. Implement Sci Commun. 2021;2(1):77. 10.1186/s43058-021-00181-2 PMC8285819 34274004

[5] Hall A, Shoesmith A, Doherty E, McEvoy B, Mettert K, Lewis CC, et al. Evaluation of measures of sustainability and sustainability determinants for use in community, public health, and clinical settings: a systematic review. Implement science: IS. 2022;17(1):81. 10.1186/s13012-022-01252-1 PMC9746194 36514059

[6] Moore JE, Mascarenhas A, Bain J, Straus SE. Developing a comprehensive definition of sustainability. Implement Sci. 2017;12(1):110. 10.1186/s13012-017-0637-1 PMC5581411 28865479

[7] Aarons GA, Hurlburt M, Horwitz SM. Advancing a conceptual model of evidence-based practice implementation in public service sectors. Adm Policy Ment Health. 2011;38(1):4–23. 10.1007/s10488-010-0327-7 PMC3025110 21197565

[8] Glasgow RE, Vogt TM, Boles SM. Evaluating the public health impact of health promotion interventions: the RE-AIM framework. Am J Public Health. 1999;89(9):1322–7. 10.2105/ajph.89.9.1322 PMC1508772 10474547

[9] NIH. Dissemination and implementation research in health program announcement (R01 Clinical Trial Optional). 2024. Available from: https://grants.nih.gov/grants/guide/pa-files/PAR-25-144.html.

[10] NIH. Dissemination and implementation research in health (R21 Clinical Trial Optional). 2024. Available from: https://grants.nih.gov/grants/guide/pa-files/PAR-25-143.html.

[11] NIH. Dissemination and Implementation Research in Health (R03 Clinical Trial Not Allowed). 2024. Available from: https://grants.nih.gov/grants/guide/pa-files/PAR-25-233.html.

[12] Neta G, Clyne M, Chambers DA. Dissemination and Implementation Research at the National Cancer Institute: A Review of Funded Studies (2006–2019) and Opportunities to Advance the Field. Cancer Epidemiol biomarkers prevention: publication Am Association Cancer Res cosponsored Am Soc Prev Oncol. 2021;30(2):260–7. 10.1158/1055-9965.EPI-20-0795 33268488

[13] Neta G, Sanchez MA, Chambers DA, Phillips SM, Leyva B, Cynkin L, et al. Implementation science in cancer prevention and control: a decade of grant funding by the National Cancer Institute and future directions. Implement Sci. 2015;10(1):4. 10.1186/s13012-014-0200-2 PMC4296529 25567702

[14] Johnson AM, Moore JE, Chambers DA, Rup J, Dinyarian C, Straus SE. How do researchers conceptualize and plan for the sustainability of their NIH R01 implementation projects? Implementation science. IS. 2019;14(1):50. 10.1186/s13012-019-0895-1 PMC6506963 31072409

[15] Norton WE, Neta G, Jacobsen PB. National Cancer Institute funding for rapid cycle interventional research in cancer care delivery. JNCI Cancer Spectr. 2023;7(6). 10.1093/jncics/pkad089 PMC10674048 37862246

[16] Page MJ, McKenzie JE, Bossuyt PM, Boutron I, Hoffmann TC, Mulrow CD, et al. The PRISMA 2020 statement: an updated guideline for reporting systematic reviews. BMJ (Clinical Res ed). 2021;372:n71. 10.1136/bmj.n71 PMC8005924 33782057

[17] The World bank country and lending groups. Available from: https://datahelpdesk.worldbank.org/knowledgebase/articles/906519-world-bank-country-and-lending-groups.

[18] Luke DA, Calhoun A, Robichaux CB, Elliott MB, Moreland-Russell S. The Program Sustainability Assessment Tool: a new instrument for public health programs. Prev Chronic Dis. 2014;11:130184. 10.5888/pcd11.130184 PMC3900326 24456645

[19] Palinkas LA, Chou C-P, Spear SE, Mendon SJ, Villamar J, Brown CH. Measurement of sustainment of prevention programs and initiatives: the sustainment measurement system scale. Implement Sci. 2020;15(1):71. 10.1186/s13012-020-01030-x PMC7470441 32883352

[20] Moullin JC, Sklar M, Ehrhart MG, Green A, Aarons GA. Provider REport of Sustainment Scale (PRESS): development and validation of a brief measure of inner context sustainment. Implement science: IS. 2021;16(1):86. 10.1186/s13012-021-01152-w PMC8404332 34461948

[21] Schell SF, Luke DA, Schooley MW, Elliott MB, Herbers SH, Mueller NB, Bunger AC. Public health program capacity for sustainability: a new framework. Implement Sci. 2013;8(1):15. 10.1186/1748-5908-8-15 PMC3599102 23375082

[22] Chambers DA, Glasgow RE, Stange KC. The dynamic sustainability framework: addressing the paradox of sustainment amid ongoing change. Implement Sci. 2013;8(1):117. 10.1186/1748-5908-8-117 PMC3852739 24088228

[23] Michie S, van Stralen MM, West R. The behaviour change wheel: a new method for characterising and designing behaviour change interventions. Implement science: IS. 2011;6:42. 10.1186/1748-5908-6-42 PMC3096582 21513547

[24] Gravitt PE, Rositch AF, Jurczuk M, Meza G, Carillo L, Jeronimo J, et al. Integrative Systems Praxis for Implementation Research (INSPIRE): an implementation methodology to facilitate the global elimination of cervical cancer. 2020;29(9):1710–9. 10.1158/1055-9965.EPI-20-0501 32561563

[25] Aarons GA, Green AE, Palinkas LA, Self-Brown S, Whitaker DJ, Lutzker JR, et al. Dynamic adaptation process to implement an evidence-based child maltreatment intervention. Implement Sci. 2012;7(1):32. 10.1186/1748-5908-7-32 PMC3436717 22512914

[26] Proctor E, Silmere H, Raghavan R, Hovmand P, Aarons G, Bunger A, et al. Outcomes for implementation research: conceptual distinctions, measurement challenges, and research agenda. Adm Policy Ment Health. 2011;38(2):65–76. 10.1007/s10488-010-0319-7 PMC3068522 20957426

[27] Iwelunmor J, Blackstone S, Veira D, Nwaozuru U, Airhihenbuwa C, Munodawafa D, et al. Toward the sustainability of health interventions implemented in sub-Saharan Africa: a systematic review and conceptual framework. Implement science: IS. 2016;11:43. 10.1186/s13012-016-0392-8 PMC4804528 27005280

[28] Shelton RC, Hailemariam M, Iwelunmor J. Making the connection between health equity and sustainability. Front Public Health. 2023;11:1226175. 10.3389/fpubh.2023.1226175 PMC10562623 37822544

[29] Hailemariam M, Bustos T, Montgomery B, Barajas R, Evans LB, Drahota A. Evidence-based intervention sustainability strategies: a systematic review. Implement Sci. 2019;14(1):57. 10.1186/s13012-019-0910-6 PMC6554955 31171004

[30] Nathan N, Powell BJ, Shelton RC, Laur CV, Wolfenden L, Hailemariam M, et al. Do the Expert Recommendations for Implementing Change (ERIC) strategies adequately address sustainment? Front Health Serv. 2022;2. 10.3389/frhs.2022.905909 PMC10012683 36925827

[31] Wiltsey Stirman S, Kimberly J, Cook N, Calloway A, Castro F, Charns M. The sustainability of new programs and innovations: a review of the empirical literature and recommendations for future research. Implement Sci. 2012;7(1):17. 10.1186/1748-5908-7-17 PMC3317864 22417162

[32] Scheirer MA, Dearing JW. An agenda for research on the sustainability of public health programs. Am J Public Health. 2011;101(11):2059–67. 10.2105/AJPH.2011.300193 PMC3222409 21940916

[33] Scheirer MA, Hartling G, Hagerman D. Defining sustainability outcomes of health programs: Illustrations from an on-line survey. Eval Program Plann. 2008;31(4):335–46. 10.1016/j.evalprogplan.2008.08.004 18835642

[34] Shediac-Rizkallah MC, Bone LR. Planning for the sustainability of community-based health programs: conceptual frameworks and future directions for research, practice and policy. Health Educ Res. 1998;13(1):87–108. 10.1093/her/13.1.87 10178339

[35] Wiltsey Stirman S, Baumann AA, Miller CJ. The FRAME: an expanded framework for reporting adaptations and modifications to evidence-based interventions. Implement science: IS. 2019;14(1):58. 10.1186/s13012-019-0898-y PMC6554895 31171014

[36] Miller CJ, Barnett ML, Baumann AA, Gutner CA, Wiltsey-Stirman S. The FRAME-IS: a framework for documenting modifications to implementation strategies in healthcare. Implement science: IS. 2021;16(1):36. 10.1186/s13012-021-01105-3 PMC8024675 33827716

