# Supplementary material for: Portfolio analysis of NCI-funded implementation science grants studying sustainability of cancer control evidence-based practices
Source: Implement Sci Commun. 2026 Aug 11;7:228. doi: 10.1186/s43058-026-01039-1 (PMC13753144; doi:10.1186/s43058-026-01039-1)
Supplement: Supplementary file 1 [file 43058_2026_1039_MOESM1_ESM.docx]

**Supplemental File 1 – Sustainability Portfolio Analysis Codebook**

| **Variable** | **Response codes & labels** |
| --- | --- |
| 1) What are the study objectives that relate to sustainability | 1. Examine factors (barriers/facilitators, context) related to sustainability 2. Evaluate the impact of a strategy on the sustainability of EBPs (what are the strategies) 3. Measure the sustainability of EBPs, strategies or practices 4. Assess costs and benefits of the sustained delivery of the evidence-based intervention, program, policy. 5. Methodological (e.g. develop measures, testing TMF or tool) 6. Other |
| 2) If other study objective, specify | Open text |
| 3) If measured, specify approach | Open text |
| 4) Area of cancer care continuum | 1. Diagnosis 2. End of life 3. Prevention 4. Screening 5. Survivorship 6. Treatment |
| 5) Cancer site | 1. Bladder cancer 2. Breast cancer 3. Cervical cancer 4. Colorectal cancer 5. Endometrial cancer 6. Kidney cancer 7. Leukemia 8. Liver cancer 9. Lung cancer 10. Lymphoma 11. Myeloma 12. Ovarian cancer 13. Prostate cancer 14. Skin cancer 15. Testicular cancer 16. Thyroid cancer 17. Other 18. Not specified |
| 6) What cancer content area does the study focus on? | 1. Comorbidity disease management (e.g., CVD; obesity) 2. Diet/Nutrition 3. Fertility 4. Financial hardship 5. Genetic services (testing, screening and counseling) 6. Patient navigation 7. Physical activity 8. Psychological well-being (e.g., depression, anxiety, quality of life) 9. Risk assessment (e.g., hereditary cancer, cervical cancer, risk-stratified screening, risk prediction models, polygenic risk scores) 10. Screening 11. Shared decision making 12. Sun Safety 13. Tobacco control 14. Social determinants of health (e.g., supportive housing, social services) 15. Survivorship care (e.g., treatment decision making, patient-caregiver social support) 16. Symptom management (e.g., pain) 17. Vaccine uptake 18. Not specified 19. Other |
| 7) If other content area specify | Open text |
| 8) Country where study is set | Open text |
| 9) Is the country a low- or middle-income country as classified by the Organisation for Economic Co-operation and Development (OECD)? <https://www.oecd.org/dac/financing-sustainable-development/development-finance-standards/daclist.htm> | 1. Yes 2. No |
| 10) At which timepoint was sustainability measure? Select all that apply | 1. 3-<6 months 2. 6-<12 months 3. 12-<18 months 4. 18-<24 months 5. 24+ months 6. Not specified |
| 11) Setting of study | 1. Health care settings (e.g., integrated delivery system, community health centers, FQHCs academic medical center) 2. Community-based organization (e.g., social service organizations, churches) 3. Health department (e.g., state, local) 4. Recreation area (e.g., park, pool, 5. School (e.g., elementary/junior/high school, college/university excluding academic medical centers) 6. Workplace (e.g., businesses, corporations) 7. Electronic (e.g. Applications) 8. Not specified 9. Other |
| 12) If other study setting, specify | Open text |
| 13) What type of study design is proposed for the sustainability objective(s) | 1. Experimental (RCT, pragmatic RCT, cluster/group RCT) 2. Mixed methods (i.e., collection and integration of qualitative and quantitative data - e.g., sequential exploratory design) 3. Observational (e.g., prospective, retrospective) 4. Qualitative (e.g., focus groups, semi-structured interviews) 5. Quasi-experimental (e.g., regression discontinuity, interrupted time series) 6. Measurement or algorithm development or validation 7. Systems science (e.g., simulation modeling, social network analysis) |
| 14) Sustainability outcomes (2) that the grant proposes to examine or measure | 1. Continuing the programs’ activities or core elements of the intervention (e.g. program implementation) 2. Maintaining community-level partnerships or coalitions and community capacity for collaboration 3. Maintaining organizational practices, procedures, and policies started during implementation (institutionalization) 4. Adaptation of intervention components 5. Not specified 6. Other |
| 15) If other outcome, specify | Open text |
| 16) Strategies being tested to increase sustainability (14, 27) | 1. Capacity building – plan for ongoing training, supervision, and/or feedback to support workforce skills. 2. Stakeholders buy-in – advocate for policy development, organize stakeholder consultations, encourage organizational or community support. 3. Consult with stakeholders (implementers, community, organizational leaders, others) strategies for sustaining the intervention/program/practice/policy after research funding is removed. 4. Monitor the effectiveness of the intervention/program/practice/policy to anticipate challenges and adapt where needed. 5. Plan for funding and/or contracting needed for continued delivery of program components. 6. Plan for implementation study to have elements of sustainability in study design (e.g. one arm includes sustainability planning) 7. None specified 8. Other |
| 17) If other strategies, specify | Open text |
| 18) At what level is context being assessed (Who is included in the interviews) | 1. Patient/consumer level 2. Provider level 3. Organization level 4. Community level 5. Policy |
| 19) Terms used | 1. Sustainability 2. Sustainment 3. Maintenance 4. Continuation 5. Not specified |
| 20) Definition used – how is sustainability defined in the grant? | Open text |
| 21) Model, theory or framework used | 1. Dynamic Sustainability Framework 2. RE-AIM 3. EPIS 4. None 5. REP 6. Framework by Shediac-Rizkallah and Bone 7. Program Sustainability Assessment Tool (PSAT) 8. Others (specify) |
| 22) If other TMF, specify | Open text |
