# Supplementary material for: Portfolio analysis of NCI-funded implementation science grants studying sustainability of cancer control evidence-based practices
Source: Implement Sci Commun. 2026 Aug 11;7:228. doi: 10.1186/s43058-026-01039-1 (PMC13753144; doi:10.1186/s43058-026-01039-1)
Supplement: Supplementary file 2 [file 43058_2026_1039_MOESM2_ESM.docx]

**Supplemental File 2 - Definitions of sustainability-related terms reported in funded applications (N=72)**

| **Sustainability Conceptualization Category^a^** | **n (%)** |
| --- | --- |
| Continued delivery or implementation of the EBP (e.g., Sustained use of the EBP over time) | 10 (13.9%) |
| Institutionalization/integration within routine practice (e.g., Extent and conditions for intervention to become institutionalized) | 8 (11.1%) |
| Adaptation and continued evolution over time (e.g., EBP adaptation to improve sustainment) | 6 (8.3%) |
| Maintenance of patient-/practice-level outcomes or behavior change (e.g., Extent to which the EBP produces the expected behavior change) | 5 (6.9%) |
| Organizational capacity or readiness for sustainability | 4 (5.6%) |
| No explicit definition provided | 39 (54.2%) |

^a^ Categories reflect the primary conceptual focus of the definition as described by investigators in the funded application. Definitions were grouped according to the main emphasis of the language used in the application rather than by reinterpretation of investigator intent. Applications using multiple terms (e.g., sustainability and maintenance) were categorized based on the primary emphasis of the definition provided, regardless of the number of terms used. Applications that referenced sustainability-related terminology without providing an explicit definition were coded as **“No explicit operational definition provided.”** To preserve confidentiality of grant application materials, definitions are presented as conceptual categories rather than verbatim text. See Table 3 for frequencies of sustainability-related terms used across funded applications.
